# Clinical Case of Metastatic Adrenocortical Carcinoma With Unusual Evolution: Review the Literature

**DOI:** 10.14740/wjon936w

**Published:** 2015-12-31

**Authors:** Luis Cabezon-Gutierrez, Ana Isabel Franco-Moreno, Parham Khosravi-Shahi, Sara Custodio-Cabello, Maria Jose Garcia-Navarro, Rosa Maria Martin-Diaz

**Affiliations:** a Department of Medical Oncology, Torrejon University Hospital, Madrid, Spain; b Department of Internal Medicine, Torrejon University Hospital, Madrid, Spain

**Keywords:** Adrenocortical carcinoma, Mitotane, Chemotherapy

## Abstract

Adrenocortical carcinoma (ACC) is a rare and heterogeneous malignancy, with an incidence of approximately 0.72 per million cases per year leading to 0.2% of all cancer deaths in the United States. Metastatic ACC has a dismal prognosis with an overall survival of less than 1 year. We present a case of a 37-year-old man with metastatic ACC with unusual good prognosis and review the therapeutic options in the literature.

## Introduction

Adrenocortical carcinoma (ACC) is a rare and heterogeneous malignancy. Adrenal tumors are very common, affecting 3-10% of the human population, and the majority are small benign non-functional adrenocortical adenomas [1]. ACC, in contrast, is a very rare disease. The Surveillance, Epidemiology, and End Results (SEER) database provides an estimation of incidence of approximately 0.72 per million cases per year leading to 0.2% of all cancer deaths in the United States [2]. The median age at diagnosis is 46 - 55 years [3]. Historically only about 30% of these malignancies are confined to the adrenal gland at the time of diagnosis. In these cases, an open resection performed by a specialized surgeon to achieve a state of no residual disease offers the best chance for cure [2]. Reoperation in case of a recurrence has been suggested to improve survival, but those studies are retrospective and may suffer from a selection bias with inclusion of patients with more indolent clinical presentations [4, 5].

Metastatic ACC has a dismal prognosis with an overall survival of less than 1 year [6]. The most common sites of metastases are the lung, liver, lymph nodes, and less commonly, the bones [2]. Palliation of functioning tumors may be achieved by resection of both the primary tumor and metastatic lesions. Unresectable or widely disseminated tumors may be palliated by adrenolytic therapy with mitotane and other antihormonal drugs (i.e., ketoconazole), and less frequently by systemic chemotherapy, and/or radiation therapy [7]. However, 5-year survival for patients with stage IV tumors is less than 20% [8, 9]. In the present communication, we describe a case in adult metastatic ACC with unusual good evolution.

## Case Report

**Figure 1:**
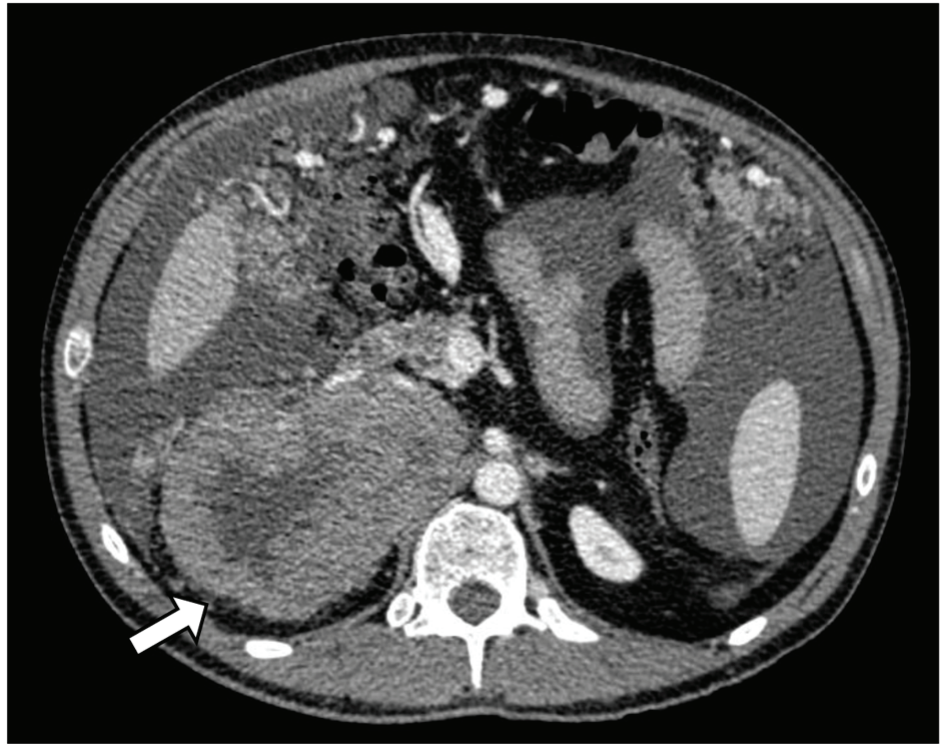
CT scan of adrenocortical carcinoma showing necrotic right adrenal mass in close proximity to the body/tail of pancreas and third portion of the duodenum (white arrow).

A 37-year-old man with no past medical history of hypertension or prior malignancy was admitted to the University Hospitals of Torrejon (Madrid, Spain) in July 2013 with worsening epigastric pain, decreased appetite, and 10 kg loss of weight over 6 months. His abdomen was mildly distended and on physical examination presented ascites. Computerized tomography (CT) (Fig. 1) revealed a huge heterogeneous adrenal tumor (13 cm in diameter) on the right side, compressing the inferior vena cava (IVC) and peritoneal carcinomatosis.

The laboratory studies did not show significant steroid hormone or catecholamine excess. A biopsy was then obtained from the adrenal mass. The tumor showed necrosis. The epithelioid component consisted of sheet and nests of loosely cohesive polygonal cells with clear and eosinophilic cytoplasm resembling adrenocortical cells. These cells showed highly atypical nuclei and large eosinophilic nucleoli. Immunohistochemical stains for AE1/AE3, S-100, calcitonin, ACTH, and chromogranin were negative. The Ki-67 proliferation index was 10%.

The patient was subsequently started on the EDP-mitotane regimen consisting of etoposide at a dose of 100 mg/m^2^ of body surface area administered intravenously on days 2, 3, and 4 of each cycle; doxorubicin at a dose of 40 mg/m^2^ given intravenously on day 1; cisplatin at a dose of 40 mg/m^2^ given intravenously on days 3 and 4; and oral mitotane administered continuously. One cycle of the regimen was defined as a 4-week interval. The patient received a total of six cycles of chemotherapy. Mitotane was started a minimum of 1 week before the initiation of the cytotoxic treatment, with the goal of attaining a blood level of 14 - 20 mg/L. Glucocorticoid replacement therapy was performed from the beginning of treatment with mitotane.

**Figure 2:**
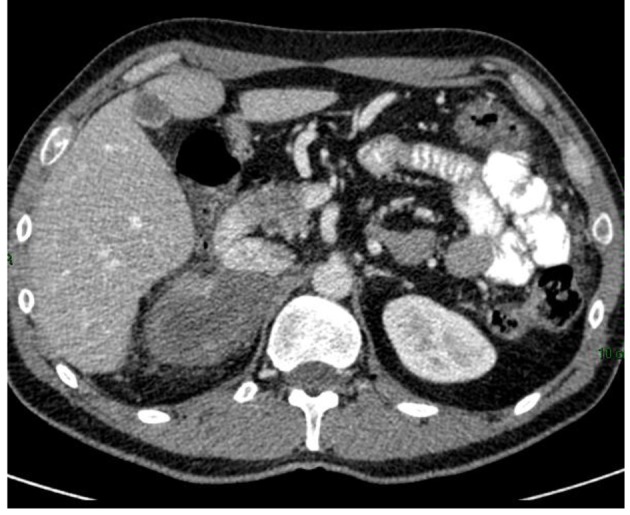
CT scan 6 months after mitotane treatment showing reduction in size of the adrenal mass.

Six months after administration of mitotane, CT scan showed a reduction in size of the adrenal mass and the peritoneal metastases (Fig. 2), with a total reduction (RECIST criteria) around 40%.

**Figure 3:**
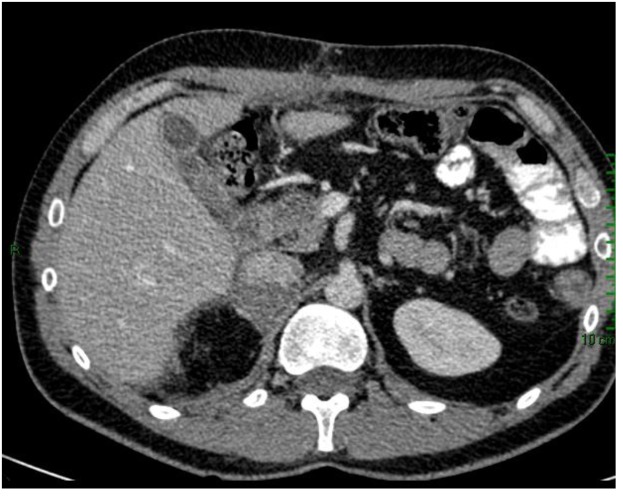
CT scan after surgical oncology showing right radical adrenalectomy and total omentectomy.

Subsequently, the patient was taken to the surgical oncology and had right radical adrenelectomy and total omentectomy. The final pathology report showed adrenocortical carcinoma, reaching surgical margins; vascular invasion; histological changes consistent with minor changes to neoadjuvant therapy; round stent without significant histological alterations; and YpT3NxM1 pathological staging. Post-surgical CT scan showed complete omentectomy and right adrenalectomy. However, an adrenal tissue around 7 × 3 cm which impresses tumor was observed. Visceral, peritoneal or bone metastatic lesions are discarded (Fig. 3).

After the operation, the patient continues treatment with mitotane, requiring frequent adjustments in dosage to maintain serum levels between 14 and 20 mg/L, and is still alive without progression 2 years after diagnosis.

## Discussion

ACC management often requires a multidisciplinary approach, frequently involving a medical oncologist, an endocrine surgeon, an endocrinologist and several other disciplines. Surgical resection remains the corner-stone of the treatment and represents the only curative option for patients with early stage ACC. However, around 80% of these patients will present local or distant recurrence after a complete resection [10]. With regard to recurrent or advanced disease, ACC is modestly responsive to standard cytotoxic chemotherapies, although various combinations have shown clear palliative benefit. Radiation and ablative techniques have been utilized with variable benefit depending on the clinical scenario. The pipeline for novel drug development and testing in clinical trials has been limited. The most frequently used systemic drugs in the advanced disease include mitotane, cisplatin and etoposide used alone or in combination with other agents [11].

Even if the tumor cannot be removed entirely, some clinicians advocate maximal debulking as a means of improving survival [12-14], although others disagree as to the survival benefit of this strategy [15, 16]. Data to support routine debulking of non-resectable tumors are lacking and decision making must be individualized, taking into account the underlying tumor biology, rate of progression, and the histologic grade [17].

Several studies confirm the prognostic value of markers of proliferation, including mitotic rate, and Ki-67 expression (as detected by a monoclonal antibody against Ki-67, MIB1) [18, 19]. The importance of proliferative rate in prognostication was shown in an analysis of 124 patients with ACC, in whom the 5-year disease-specific survival rates for individuals with tumor mitotic rates of ≤ 5, 6 - 10, 21 - 50, and > 50 per 50 high-power fields were 63%, 50%, 25%, and 0%, respectively [18]. Malandrino et al demonstrated in the multivariate analysis like mitotane level 14 mg/L and objective response to platinum-based chemotherapy were found to be independent predictors of survival (P < 0.03 and P < 0.001). This study suggests a prognostic role for mitotane therapy and objective response to platinum-based chemotherapy [20].

Evidence for use of mitotane as the only approved drug for advanced ACC is based on retrospective series of data as opposed to any prospective, randomized controlled trials. Mitotane is considered one of the most active agents in ACC with response rates ranging from 13% to 31% [21]. However, complete responses rarely occur. In terms of overall survival, four studies concluded that mitotane treatment does not increase the survival rate whereas five found an increase in the survival rate [13, 21-23].

Primary treatment with mitotane may be indicated for patients who have histologically proven ACC in whom surgery is incomplete, not feasible, or contraindicated. The quality of the available literature on mitotane monotherapy is poor, and the results are highly variable. Most studies were conducted in early years without monitoring of tumor response with adequate imaging. It is generally accepted, though not proven, that chemotherapy plus mitotane produces better outcomes than does mitotane alone. Therefore, in the setting of extensive (multiple tumor-involved organs) rapidly progressive high-grade disease, mitotane is almost always administered in combination with cytotoxic chemotherapy. A few prospective trials have explored combination regimens that contain mitotane (Table 1) [24-27].

**Table 1:** Cytotoxic Chemotherapy Plus Mitotane in ACC [24-27]

Drug	Study phase/n	Clinical benefit
Cisplatin + etoposide followed by mitotane [24]	II/47	ORR: 11% Median OS: 10 months
M/EDP [25]	II/28	ORR: 53.5% CR: two patients PR: 13 patients
SM [26]	II/22	ORR: 36.4%
M/EDP vs. SM [27]	III/304	ORR: 23.2 vs. 9.2% PFS: 5.0 vs. 2.1 months OS: 14 vs. 12 months

M/EDP: mitotane + etoposide, doxorubicin and cisplatin; SM: streptozotocin + mitotane.

Although no chemotherapy regimen has been shown to improve overall survival in patients with advanced ACC, some of the more encouraging results have been with the combination of EDP plus mitotane. The largest trial of advanced ACC to date, the First International Randomized trial in locally advanced and Metastatic Adrenocortical Carcinoma Treatment (FIRM-ACT), randomly assigned 304 patients with advanced ACC not amenable to radical surgery to mitotane plus either EDP or streptozotocin [27]. Rates of objective tumor response (23% versus 9%) and median progression-free survival (PFS) (5 versus 2.1 months) were both significantly better in the EDP-mitotane (EDP-M) group, although these benefits did not translate into a significantly prolonged survival (median 14.8 versus 12 months). This lack of significant difference in overall survival, despite better PFS for EDP-M, is possibly due to the cross-over design of the study, since EDP-M was superior as a second-line treatment as well. Rates of serious adverse events did not differ significantly between treatments. The efficacy of both regimens as second-line therapy was similar to their efficacy as first-line therapy. Results with other chemotherapy drugs combined with mitotane seem to be less promising [26-29].

Because of the age of our patient, low Ki-67 (< 10%), good response to chemotherapy and good performance status (0), we decided to perform debulking surgery and subsequently maintaining mitotane therapy. From the date of diagnosis of the disease, it has not been objectified progression, with a PFS of 24 months, which exceeds the usual median of 5 months [27]. This difference is probably explained by the patient’s age, good performance status, Ki-67 < 10% and the possibility of debulking surgery.

A better understanding of ACC biology has provided some rationale for the development of novel agents in this disease. Unfortunately, new agents have resulted in minimal or no activity and new investigations/clinical trials need to be encouraged to incorporate this strategy in the clinical practice (Table 2) [30-41].

**Table 2:** Cytotoxic Chemotherapy in ACC [31-41]

Drug	Target	Study phase/n	Clinical benefit
Sunitinib [32]	VEGF pathway	II/35	Five patients with SD
Sorafenib + paclitaxel [33]	VEGF pathway + chemotherapy	II/9	No benefit
Bevacizumab + capecitabina [34]	VEGF pathway + chemotherapy	II/10	No benefit
Erlotinib + gemcitabina [35]	EGFR pathway + chemotherapy	II/10	One patient with SD
Gefitinib [36]	EGFR pathway	II/19	No benefit
Figitumumab [37]	IGF-1R pathway	II/14	Eight patients with SD
Cixutumumab [38]	IGF-1R pathway	II/10	One patient with SD
Cixutumumab + temsirolimus [39]	IGF-1R pathway + mTOR pathway	I/10	Four patients with SD
Everolimus [40]	mTOR pathway	II/4	No benefit
Imatinib [41]	c-ABL, PDGFR and c-Kit pathway	II/4	No benefit
Linsitinib [31]	Dual inhibitor of IGFR and IR tyrosine kinase	III/139	No benefit

VEGF: vascular endothelial growth factor; EGFR: epidermal growth factor receptor; IGF-1R: insulin growth factor 1 receptor; mTOR: mammalian target of rapamycin; PDGFR: platelet-derived growth factor receptor.

A placebo-controlled phase III trial of linsitinib, an oral small molecule of both the IGF-1R and insulin receptor, in advanced ACC failed to demonstrate any benefit in disease-free or overall survival [31]. However, as with cixutumumab, partial responses and disease stabilization (in some cases lasting for years) were seen in a small subset of patients. Although IGF-1R inhibitors cannot yet be recommended to any patient with advanced ACC, ongoing efforts to determine the genetic fingerprint that defines it will hopefully allow future selection of patients who might benefit from this approach.

The role of immune regulation in tumor progression and metastasis has been investigated and the interest in this subject is tremendously increasing. As an example, blocking programmed death-1 (PD-1), an inhibitory receptor expressed on activated T cells, results in significant antitumor activity in several malignancies. Efforts should be also done to clarify the potential of novel immunotherapies to treat ACC and at least to interrogate the expression of the PD-1 ligand on tumor cells [30].

### Conclusion

The outcomes of the patient reported here with survival greater than 24 months argue for an aggressive treatment approach using a multidisciplinary team to discuss treatment options including chemotherapy, surgery and mitotane when feasible.
